# Effects of aerobic, resistance, and combined training on endothelial function and arterial stiffness in older adults: study protocol for a systematic review and meta-analysis

**DOI:** 10.1186/s13643-022-02036-w

**Published:** 2022-08-13

**Authors:** Raphael S. N. da Silva, Diego S. da Silva, Gustavo Waclawovsky, Maximiliano I. Schaun

**Affiliations:** Instituto de Cardiologia do Rio Grande do Sul/Fundação Universitária de Cardiologia, Av. Princesa Isabel, 395 Santana, Porto Alegre, RS 90620-001 Brazil

**Keywords:** Exercise, Endothelium, Reactive hyperemia, Vascular stiffness, Pulse wave analysis, Aging

## Abstract

**Introduction:**

Aging is an independent risk factor for cardiovascular events. It promotes vascular dysfunction which is associated with risk factors for cardiovascular diseases (CVDs). Exercise can modulate vascular function parameters, but little is known about the effects of different modalities of training (aerobic, resistance, and combined) on endothelial function and arterial stiffness in older adults.

**Methods:**

This systematic review study will include randomized controlled trials (RCTs) selected from the electronic databases MEDLINE (PubMed), Cochrane, LILACS, EMBASE, and Web of Science. We will follow the PRISMA guidelines and PICOS framework. Studies involving both male and female older adults (≥60 years old) with or without comorbidities undergoing aerobic, resistance, and/or combined training compared to a control group (no exercise) will be eligible. We will use the Cochrane Risk of Bias 2 (RoB 2) tool to evaluate the quality of individual studies and GRADE to assess the strength of evidence. Statistical analyses will be conducted with RStudio for Windows (v1.3.959) using R package meta.

**Discussion:**

A systematic review and meta-analysis involving data from studies of older adults would deepen our understanding of vascular adaptations to exercise training in this population. It could provide new insights into how health providers can improve patient management and prevention of cardiovascular events in older adults.

**Systematic review registration:**

PROSPERO 42021275451

## Introduction

Aging is a major non-modifiable risk factor for the development of cardiovascular diseases (CVDs) and mortality [1, 2]. Age-related pathophysiological conditions associated with CVDs are due to alterations of vascular structure (arterial stiffness) and function and can lead to an imbalance of protective mechanisms against hemodynamic fluctuations and thus to vascular dysfunction [3–7].

Vascular dysfunction is an independent risk factor for CVDs associated with atherosclerotic pathophysiological alterations [8–10] as well as several other risk factors such as hypertension [11–13], diabetes melitus [14, 15], chronic kidney disease [16–18], obesity [19, 20], dysplipidemia [21], metabolic syndrome [22], physical inactivity [23–25], and even forms of cognitive impairment such as dementia and Alzheimer’s disease [26–29].

Endothelial function and arterial stiffness are considered important biomarkers of vascular function and predictors of cardiovascular events that are more accurate than well-established traditional risk factor scores [6, 10, 30–35]. Furthermore, epidemiological studies have demonstrated that endothelial dysfunction and increased arterial stiffness are highly prevalent among older adults [4, 36–38]. Therefore, the aging is an independent risk factor associated with vascular dysfunction, which increases the risk for the development of CVDs [2, 4, 32, 38–41].

Flow-mediated dilation (FMD) and pulse wave velocity (PWV) have been widely used in clinical practice and research studies to assess endothelial function and arterial stiffness, respectively [35, 42–46]. Several meta-analyses have shown that every 1% increase in FMD is associated with an 8–16% lower risk of fatal and non-fatal CV events and/or deaths from all causes with even greater effects in individuals with established CVDs [47–50]. Similarly, a 1m/s increase of PWV has been associated with up to 15% increase in the risk of CVD death [51, 52].

Different exercise training modalities (aerobic, resistance, and combined) have been recommended to reduce morbidity and mortality from CVDs and modify CVD risk profiles [53–64]. However, exercise training modalities and their variables (exercise volume, frequency, intensity, order, etc.) are not all similarly effective and may elicit different beneficial effects on vascular function parameters [65–70]. A few studies of resistance training has reported null [71–75] or even negative findings [76, 77] for the effect on arterial stiffness and endothelial function [74, 75], but they relate to healthy young and middle-aged adults [71, 73–76] and adults with comorbidities [70–72, 77]. In a recent meta-analysis conducted by our group [78] evaluating the effect of aerobic training in individuals with hypertension (362; aged 52 to 67 years), we found a 1.45% increase (*p*=0.001) in FMD, though we considered this hypothesis null (95% CI –0.11 to 3.00). On the other hand, randomized controlled trials (RCTs) and meta-analyses conducted by our group [78, 79] and other authors [80, 81] have found positive effects of different training modalities on endothelial function assessed by FMD. Yet, similar effects on arterial stiffness have not been demonstrated with resistance and combined training [67, 69, 82, 83]. Sample heterogeneity (healthy individuals or individuals with comorbidities) and predominant representation of specific age groups (often young and/or middle-aged adults) are some issues found in several meta-analyses, which shows that this subject has been little explored in older adults. Therefore, a systematic review and meta-analysis of RCTs is necessary to determine whether exercise training (aerobic, resistance, or combined) exert an effect on endothelial function and arterial stiffness in older adults.

## Methods

**Fig. 1:**
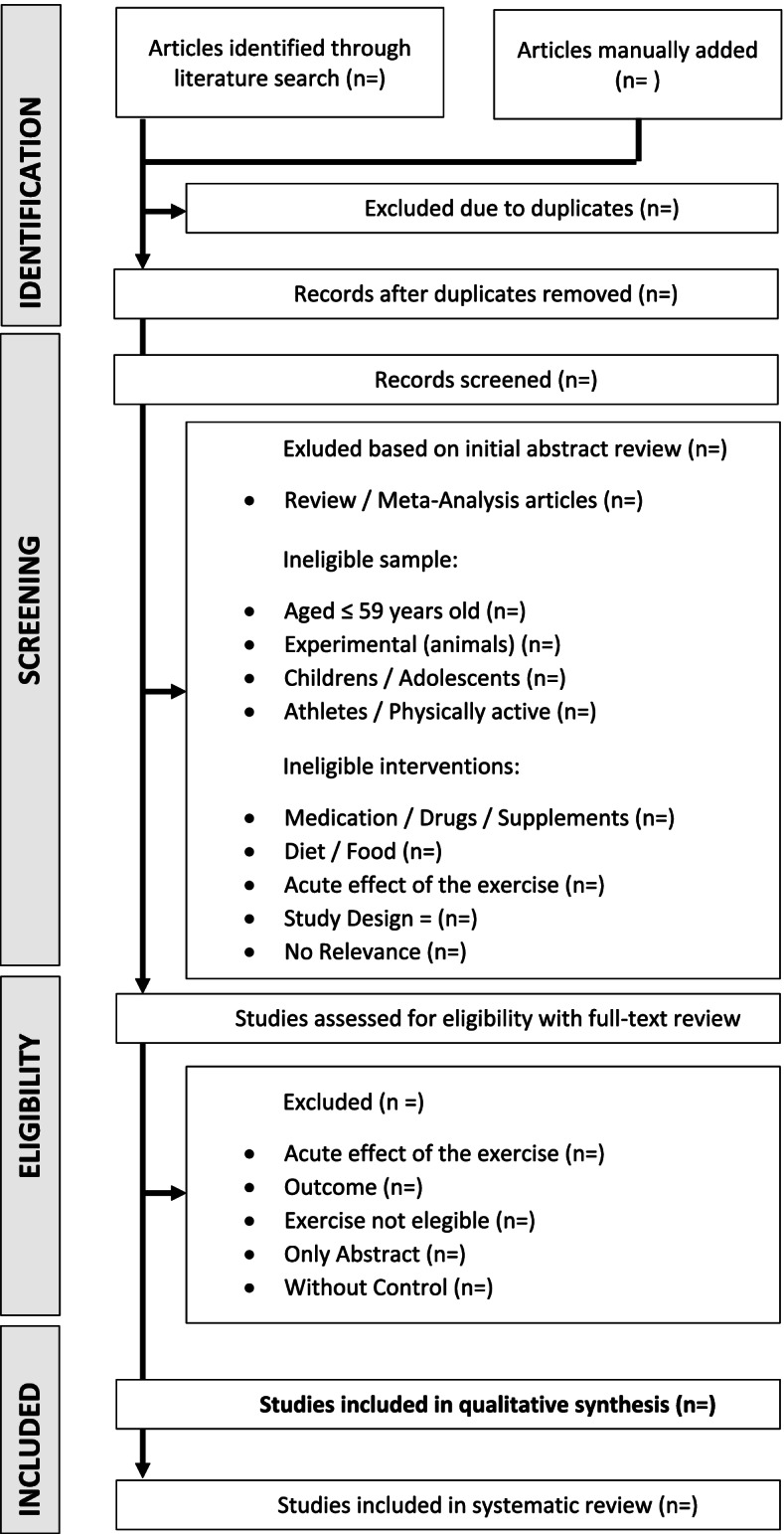
Flowchart of the study design

Our systematic review will follow the guidelines of the Preferred Report Items for Systematic Reviews and Meta-Analysis (PRISMA) [84] and the specific PRISMA-P guidelines [85]. This protocol for a systematic review and meta-analysis was registered in the International Prospective Register of Systematic Reviews (PROSPERO) (www.crd.york.ac.uk/PROSPERO/, ID 42021275451, registered on 29 September 2021). For the sake of research transparency, the database used in the systematic review and meta-analysis will be made available on Mendeley Data repository as open access (https://data.mendeley.com/). Figure 1 summarizes the flowchart of the study design.

### Eligibility criteria

We used PICOS as a framework to develop the study design, research questions, and searches for our systematic review as follows: **P**opulation (individuals aged ≥ 60 years), **I**ntervention (aerobic and/or resistance and/or combined exercise training), **C**omparison (training group vs. control group [no exercise]), **O**utcome (endothelial function assessed by FMD and arterial stiffness assessed by PWV), and **S**tudy (RCTs).

The inclusion criteria for studies in this systematic review are (1) direct evidence from RCTs comparing one or more interventions (aerobic, resistance, or combined training) to a control group (no exercise training, usual care, education, placebo, and/or waiting list for intervention); (2) 4 weeks or more of intervention; (3) assessment of endothelial function and/or arterial stiffness as the primary or secondary outcome; (4) study population of both male and female adults aged ≥ 60 years; and (5) no language restrictions.

Since our systematic review will involve physical activity and its variables, we will adopt the following conceptual definitions for the searches: **(I) physical activity**: any bodily movement produced by skeletal muscles that results in energy expenditure, but it does not require or imply any specific aspect or quality of movement, and it may encompass work, leisure, recreational, sports, and fitness activities among others [86, 87]; **(II) exercise**: a subgroup of physical activity that is planned, structured, repetitive (i.e., exercise training) and designed to improve or maintain one or more components of physical fitness, physical performance, and health [86, 87]; **(III) aerobic training**: any form of activity that predominantly uses the aerobic energy system involving large muscle groups in a rhythmic manner for a sustained period of time to maintain or improve cardiorespiratory fitness [62, 87]; **(IV) resistance training**: any form of activity that involves voluntary, repeated and short muscle contractions, either dynamic, or isometric, carried out against an external resistance (load) that is greater than that usually found in activities of daily living to maintain or increase muscular strength, power, endurance, and mass [87, 88]; and **(V) combined training**: a combination of aerobic plus resistance exercises (in any order) carried out in the same session or on alternate days of the week designed to improve both aerobic fitness and muscle strength [89, 90].

### Search strategy

We will conduct searches in five databases: (1) MEDLINE (via PubMed), (2) LILACS, (3) Embase, (4) Cochrane Central Register of Controlled Trials (CENTRAL), and (5) Web of Science. We will evaluate articles of similar subjects included in previous reviews to avoid missing any potential eligible studies. To minimize any publication bias, we will also conduct searches on online gray literature including OpenGrey (www.opengrey.eu) and the Brazilian Coordination for the Improvement of Higher Education Personnel (CAPES) Bank of Theses and Dissertations (www.catalogodeteses.capes.gov.br). For unpublished ongoing studies, our searches will be undertaken in the following clinical trial registries: Brazilian Clinical Trials Registry (ReBEC, www.ensaiosclinicos.gov.br), ClinicalTrial.gov (www.clinicaltrials.gov), and WHO International Clinical Trials Registry Platform (ICTRP, www.who.int/ictrp/en/). We will collect data through careful review of the retrieved articles, and the authors will be contacted by email to obtain any additional information if needed.

Our reviewers (RSNS and GW) will create specific search strategies using Boolean operators (AND; OR) for each database (Table 1). MeSH and entry terms will primarily follow those available in PubMed, and they will be adjusted to each database. The following key terms will be used: “exercise,” “resistance training,” “endurance training,” “vascular endothelium,” and “vascular stiffness”. To increase accuracy and sensitivity of our search, the terms for RCTs in the MEDLINE [91] and EMBASE [92] databases will be added to the search terms.

**Table 1:** Search strategy

**MEDLINE (PubMed)**	
***((EXERCISE [MeSH Terms]****OR Exercises OR Physical Activity OR Activities, Physical OR Activity, Physical OR Physical Activities OR Exercise, Physical OR Exercises, Physical OR Physical Exercise OR Physical Exercises OR Acute Exercise OR Acute Exercises OR Exercise, Acute OR Exercises, Acute OR Exercise, Isometric OR Exercises, Isometric OR Isometric Exercises OR Isometric Exercise OR Exercise, Aerobic OR Aerobic Exercise OR Aerobic Exercises OR Exercises, Aerobic OR Exercise Training OR Exercise Trainings OR Training, Exercise OR Trainings, Exercise)****OR (Resistance Training [MeSH Terms]****OR Training, Resistance OR Strength Training OR Training, Strength OR Weight-Lifting Strengthening Program OR Strengthening Program, Weight-Lifting OR Strengthening Programs, Weight-Lifting OR Weight Lifting Strengthening Program OR Weight-Lifting Strengthening Programs OR Weight-Lifting Exercise Program OR Exercise Program, Weight-Lifting OR Exercise Programs, Weight-Lifting OR Weight Lifting Exercise Program OR Weight-Lifting Exercise Programs OR Weight-Bearing Strengthening Program OR Strengthening Program, Weight-Bearing OR Strengthening Programs, Weight-Bearing OR Weight Bearing Strengthening Program OR Weight-Bearing Strengthening Programs OR Weight-Bearing Exercise Program OR Exercise Program, Weight-Bearing OR Exercise Programs, Weight-Bearing OR Weight Bearing Exercise Program OR Weight-Bearing Exercise Programs)****OR (Endurance Training [MeSH Terms]****OR Training, Endurance****)) AND ((Vascular Endothelium [MeSH Terms]****OR Endothelium, Vascular OR Endotheliums, Vascular OR Vascular Endotheliums OR Capillary Endothelium OR Capillary Endotheliums OR Endothelium, Capillary OR Endotheliums, Capillary)****OR (Vasodilation [MeSH Terms]****OR Vasorelaxation OR Vasodilatation OR Vascular Endothelium-Dependent Relaxation OR Endothelium-Dependent Relaxation, Vascular OR Relaxation, Vascular Endothelium-Dependent OR Vascular Endothelium Dependent Relaxation)****OR (Hyperemia [MeSH Terms] OR****Hyperemias OR Active Hyperemia OR Hyperemia, Active OR Arterial Hyperemia OR Hyperemia, Arterial OR Venous Engorgement OR Engorgement, Venous OR Venous Congestion OR Congestion, Venous OR Passive Hyperemia OR Hyperemia, Passive OR Reactive Hyperemia OR Hyperemia, Reactive OR Hyperemias, Reactive OR Reactive Hyperemias)****AND (Vascular Stiffness [MeSH Terms] OR****Vascular OR Vascular Stiffnesses OR Arterial Stiffness OR Arterial Stiffnesses OR Stiffness, Arterial OR Aortic Stiffness OR Aortic Stiffnesses OR Stiffness, Aortic)****OR (Pulse Wave Analysis [MeSH Terms]****OR Analyses, Pulse Wave OR Analysis, Pulse Wave OR Pulse Wave Analyses OR Wave Analyses, Pulse OR Wave Analysis, Pulse OR Pulse Wave Velocity OR Pulse Wave Velocities OR Velocities, Pulse Wave OR Velocity, Pulse Wave OR Wave Velocities, Pulse OR Wave Velocity, Pulse OR Pulse Transit Time OR Pulse Transit Times OR Time, Pulse Transit OR Times, Pulse Transit OR Transit Time, Pulse OR Transit Times, Pulse OR Pulse Wave Transit Time****)) AND (RANDOMIZED CONTROLLED TRIAL[pt]***OR controlled clinical trial[pt] OR randomized controlled trials[mh] OR random allocation[mh] OR double-blind method[mh] OR single-blind method[mh] OR clinical trial[pt] OR clinical trials[mh] OR “clinical trial”[tw] OR singl*[tw] OR doubl*[tw] OR trebl*[tw] OR tripl*[tw] OR random*[tw] OR cross-over studies[mh] OR control*[tw] OR volunteer*[tw])	
**COCHRANE**	
*((Exercise) OR (Physical Activity) OR (Training)) AND ((Endothelium) OR (Vasodilation) OR (Hyperemia)) AND ((Vascular Stiffness) OR (Arterial Stiffness) OR (Pulse Wave Analysis))*	
**Web Of Science**	
*TS=(exercise OR training OR physical activity) AND TS=(endothelium OR vasodilation OR vasorelaxation OR hyperemia) AND TS=(vascular OR arterial OR pulse wave velocity OR sitffness) AND TS=(randomized controlled trial OR randomized controlled trial)*	
**EMBASE**	
*('exercise'/exp OR 'exercise' OR 'training'/exp OR 'training' OR 'physical activity'/exp OR 'physical activity') AND ('vascular endothelium'/exp OR 'vascular endothelium' OR 'hyperemia'/exp OR 'hyperemia' OR 'vasodilatation'/exp OR 'vasodilatation') AND (vascular OR 'arterial stiffness'/exp OR 'arterial stiffness' OR 'pulse wave'/exp OR 'pulse wave') AND ('randomized controlled trial'/exp OR 'randomized controlled trial')*	
**LILACS (Portuguese)**	
*(tw:(****Exercício Físico****)) AND (tw****:(Endotélio****)) OR (tw:(****Endotélio Vascular****)) OR (tw:(****Vasodilatação****)) OR (tw:(****hiperemia****)) AND (tw:(****Rigidez vascular****)) OR (tw:(****Análise de Onda de Pulso****))*	
**LILACS (Spanish)**	
*(tw:(****Ejercicio físico****)) AND (tw****:(Endotelio****)) OR (tw:(****Endotelio Vascular****)) OR (tw:(****Vasodilatación****)) OR (tw:(****hiperemia****)) AND (tw:(****Rigidez vascular****)) OR (tw:(****Análisis de la onde del pulso****))*	
**LILACS (English)**	
*(tw:(****Exercise****)) AND (tw****:(Endothelium****)) OR (tw:(****Endothelium, Vascular****)) OR (tw:(****Vasodilation****)) OR (tw:(****hyperemia****)) AND (tw:(****Vascular Stiffness****)) OR (tw:(****Pulse Wave Analysis****))*	

For selection of eligible studies, all articles retrieved will be saved as “.ris” and imported into EndNote (v.X9, Thomson-Reuters*;* 2019, New York, USA) and arranged in folders (by database, inclusion criteria, and exclusion criteria). Two independent reviewers (RSNS and DSS) will screen all articles based on their titles and abstracts. The reasons for excluding studies will be categorized as follows: ineligible population (adults aged ≤ 59 years; children and/or adolescents); ineligible intervention (alternative forms of exercise including martial arts, Tai Chi, Yoga, exercise for relaxation, meditation, stretching, vibration machine exercises, and/or muscle electrostimulation) and no details and FITT [Frequency, Intensity, Time and Type]) information available; animal studies or in vitro experimentation; exercise along with dietary interventions, nutritional supplements, and/or drug use; outcomes of interest assessed by techniques other than FMD or PWV; and ineligible study designs (cohort, observational, case-control and case report studies, reviews and protocols). Any disagreements will be resolved by a third reviewer (MIS or GW).

### Data extraction and management

Our reviewers (RSNS and DSS) will read separately the full text of all eligible studies. If a study is relevant for inclusion in the review, each reviewer will manually extract and compile the main data in a pre-structured database in Excel 365 for Windows. For the extraction of data from RCTs with the outcomes of interest presented in graphs, we will use WebPlotDigitizer to extract the data (https://apps.automeris.io/wpd/). For studies assessing changes in endothelial function or arterial stiffness at different time points after the intervention, we will independently compare baseline measurements with results at each time point. For studies assessing arterial stiffness with different PWV indices, we will prioritize central PWV (carotid-femoral/carotid PWV) [46, 51] over peripheral PWV measurements brachial-ankle/femoral-ankle PWV) [44, 45].

The data extracted will be divided into five main groups: study identification (authors and year of publication), participants (age, gender, and medical conditions), material and methods (randomization, blinding, and sample size), intervention (FITT components), secondary outcomes of interest (anthropometric measures, biochemical data, oxygen consumption, strength level), and techniques (FMD and PWV detailed description: location of the measurement) [45, 93].

### Risk of bias

The risk of bias of eligible studies will be assessed using Cochrane Risk of Bias 2 (RoB) 2 tool [94]. The assessment is based on a set of six domains of bias: (1) randomization process, (2) deviations from intervention (allocation concealment sequence), (3) incomplete outcome data, (4) outcome assessment, (5) selective reporting, and (6) absolute bias. Based on that, the studies will be classified as low risk of bias (in all domains for this result), some concerns of bias (in at least one domain, but not at high risk of bias in any domain), or high risk of bias (in at least one domain for this result or the study was judged to be at some concerns for several domains in a way that significantly reduces confidence in the outcome). Since it is not possible to blind participants to exercise training interventions, all studies will be classified as high risk of bias in the domain “deviations from intervention.”

### Quality of evidence

The strength of the body of evidence will be assessed using the GRADE (Grading of Recommendations, Assessment, Development and Evaluation) tool (www.gradeworkinggroup.org/) [95, 96]. This tool classifies the quality of evidence into four levels (high, moderate, low, and very low) based on the assessment of confidence in specific estimates in five domains: methodological limitations (risk of bias), inconsistency, indirectness of evidence, imprecision, and publication bias.

### Statistical analysis

The outcomes of interest are changes in FMD and PWV in response to exercise training compared to the control group. We will calculate the difference (delta) of absolute values between post and pre-training in both the intervention group and the control group. We will use the same procedure to calculate the standard deviations (SDs).

All measures of effect will be presented as mean differences (MDs) between training versus control groups, and their related 95% confidence intervals (95% CIs). If the studies do not have sufficient similarities to warrant a fixed-effects model, the mean differences will be pooled using a random-effects model. Since the 95% CI from random effects refer to uncertainty in the location of the mean of systematically different effects in the studies, we will consider the calculated values for a prediction interval (PI) as they reflect the interval of uncertainty of the effects to be expected in future RCTs [97].

To assess the consistency of training effect among studies, the degree of heterogeneity (relative variability in effect estimates attributed to heterogeneity) will be tested using the Higgins inconsistency test (*I*^2^) for every pairwise comparison [98, 99] (Table 2). To explore the heterogeneity (*p*<0.05), we will conduct (observational) subgroup analyses and/or meta-regression (statistics; ≥10 studies) for effect modifiers with normal distribution in a quartile-quartile plot (qq-plot) and confirm it with the Shapiro-Wilk test (*p*>0.05) [100]. In addition, to remove discrepant data from the meta-analysis, forest plots will be constructed to visualize the effect estimate of individual studies and detect outliers based on non-CI overlapping that is due to heterogeneity [100]. If there is significant heterogeneity between studies that cannot be explained, we will not perform a meta-analysis and estimates of intervention effects from the studies selected will be presented individually instead.

**Table 2:** Interpretation of heterogeneity results

Interpretation of *I*^2^	
0 to 40%: might not be important	
30 to 60%: may represent moderate heterogeneity^a^	
50 to 90%: may represent substantial heterogeneity^a^	
75 to 100%: considerable heterogeneity^a^	

^a^The importance of the value of *I*^2^ depends on the magnitude and direction of effects and the strength of evidence for heterogeneity (*I*^*2*^ confidence interval: uncertainty of the value of *I*^2^ is substantial when there is a small number of studies)

Potential effect modifiers, including age, body mass index (BMI), baseline FMD, baseline arterial stiffness indices (intervention and control), and FITT components will be analyzed separately. If applicable (≥ 10 studies), we will perform the Egger’s test using a funnel plot to assess potential publication bias in the meta-analysis.

All statistical tests will be two-tailed and the significance level will be set at *p*<0.05. All measures of dispersion presented as CIs or standard errors will be converted into SDs before the meta-analysis. Alternative analyses to the primary analysis, including a sensitivity analysis, could be performed to determine the robustness of our decisions (i.e., missing value imputation method used, inclusion of studies with high risk of bias, data from conference abstracts and others) [101].

Data modelizations will be performed with RStudio (version 1.3.959) using the R package meta (version 3.6.1) for Windows (https://www.r-project.org/). A RStudio script for conducting the meta-analysis is shown in Table 3.

**Table 3:** Script used for the meta-analysis of data from systematic review

• library (readxl) • FMD_RV <- read_excel ("C:/Metanalysis_Raphael/database_analysis/FMD_RV.xlsx") • View (FMD_RV) • Meta_1 = FMD_RV <- metacont (t_n, t_mean, t_dp, c_n, c_mean, c_dp, Study, predict = TRUE, data = FMD_RV, sm = "MD") • Meta_1 • forest (Meta_1, sortvar = Study, xlim = c (-10.0, 10.0), predict = TRUE, col.square = "grey", col.diamond = "black", digits = 2) • forest (Meta_1, comb.fixed = FALSE, sortvar = Study, xlim = c (-10.0, 10.0), digits.sd = 2, digits.I2= 2, print.I2.ci = TRUE , digits.tau2 = 2, digits.pval.Q = 3, squaresize = 0.5, lab.e = "Experimental", lab.c = "Control", col.inside = "black", col.square = "grey", col.diamond = "black", col.predict = "transparent", digits = 2) • baujat (Meta_1, ylim = c (-1.0, 1.0), xlim = c (-200, 200)) • metainf (Meta_1, pooled = "random") • metabias (Meta_1, method.bias = "linreg") • funnel (meta_Rapha1)	

## Discussion and conclusions

Different modalities of exercise training appear to improve or at least do not cause harm to endothelial function in different populations [68, 78, 80, 81]. In contrast, resistance and combined exercise training exert null [69, 83] or even negative effects [70, 76, 77] on different of vascular function parameters especially arterial stiffness. These findings may be associated with strength training variables and heterogeneity of the populations studied [66, 69, 102]. Given that aging is an independent risk factor for CVDs [1, 2, 40], a systematic review may help further understand the changes elicited in vascular structure and function from interventions with different modalities of exercise training in older people.

## Data Availability

Not applicable.

## References

[1] Costantino S, Paneni F, Cosentino F (2016). Ageing, metabolism and cardiovascular disease. J Physiol.

[2] Paneni F, Diaz Cañestro C, Libby P, Lüscher TF, Camici GG (2017). The aging cardiovascular system: understanding it at the cellular and clinical levels. J Am Coll Cardiol.

[3] Brandes RP, Fleming I, Busse R (2005). Endothelial aging. Cardiovasc Res.

[4] Mitchell GF, Wang N, Palmisano JN, Larson MG, Hamburg NM, Vita JA (2010). Hemodynamic correlates of blood pressure across the adult age spectrum: noninvasive evaluation in the Framingham Heart Study. Circulation.

[5] Van Sloten TT (2017). Vascular dysfunction: at the heart of cardiovascular disease, cognitive impairment and depressive symptoms. Artery Res.

[6] Correia ML, Haynes WG (2007). Arterial compliance and endothelial function. Curr Diabetes Rep.

[7] Gerhard M, Roddy MA, Creager SJ, Creager MA (1996). Aging progressively impairs endothelium-dependent vasodilation in forearm resistance vessels of humans. Hypertension (Dallas, Tex: 1979).

[8] Bonetti PO, Lerman LO, Lerman A (2003). Endothelial dysfunction: a marker of atherosclerotic risk. Arterioscler Thromb Vasc Biol.

[9] Gimbrone MA, García-Cardeña G (2016). Endothelial cell dysfunction and the pathobiology of atherosclerosis. Circ Res.

[10] Zieman SJ, Melenovsky V, Kass DA (2005). Mechanisms, pathophysiology, and therapy of arterial stiffness. Arterioscler Thromb Vasc Biol.

[11] Soloviev MA, Kulakova NV, Semiglazova TA, Borodulina EV, Udut VV (2011). Correction of endothelial dysfunction in patients with arterial hypertension. Bull Exp Biol Med.

[12] Perticone F, Ceravolo R, Pujia A, Ventura G, Iacopino S, Scozzafava A (2001). Prognostic significance of endothelial dysfunction in hypertensive patients. Circulation.

[13] Blacher J, Asmar R, Djane S, London GM, Safar ME (1999). Aortic pulse wave velocity as a marker of cardiovascular risk in hypertensive patients. Hypertension (Dallas, Tex: 1979).

[14] Abebe W, Mozaffari M (2010). Endothelial dysfunction in diabetes: potential application of circulating markers as advanced diagnostic and prognostic tools. EPMA J.

[15] Tynjälä A, Forsblom C, Harjutsalo V, Groop PH, Gordin D (2020). Arterial stiffness predicts mortality in individuals with type 1 diabetes. Diabetes Care.

[16] Blacher J, Guerin AP, Pannier B, Marchais SJ, Safar ME, London GM (1999). Impact of aortic stiffness on survival in end-stage renal disease. Circulation.

[17] Guerin AP, Blacher J, Pannier B, Marchais SJ, Safar ME, London GM (2001). Impact of aortic stiffness attenuation on survival of patients in end-stage renal failure. Circulation.

[18] London GM, Marchais SJ, Safar ME, Genest AF, Guerin AP, Metivier F (1990). Aortic and large artery compliance in end-stage renal failure. Kidney Int.

[19] Iantorno M, Campia U, Di Daniele N, Nistico S, Forleo GB, Cardillo C (2014). Obesity, inflammation and endothelial dysfunction. J Biol Regul Homeost Agents.

[20] Recio-Rodriguez JI, Gomez-Marcos MA, Patino-Alonso MC, Agudo-Conde C, Rodriguez-Sanchez E, Garcia-Ortiz L (2012). Abdominal obesity vs general obesity for identifying arterial stiffness, subclinical atherosclerosis and wave reflection in healthy, diabetics and hypertensive. BMC Cardiovasc Disord.

[21] Lind L (2002). Lipids and endothelium-dependent vasodilation--a review. Lipids.

[22] Vykoukal D, Davies MG (2011). Vascular biology of metabolic syndrome. J Vasc Surg.

[23] Pahkala K, Heinonen OJ, Simell O, Viikari JS, Rönnemaa T, Niinikoski H (2011). Association of physical activity with vascular endothelial function and intima-media thickness. Circulation.

[24] Laslovich S, Alvar BA, Allison M, Rauh MJ (2020). Effects of lifestyle physical activity on vascular function in asymptomatic peripheral arterial disease. Med Sci Sports Exerc.

[25] Endes S, Schaffner E, Caviezel S, Dratva J, Autenrieth CS, Wanner M (2016). Physical activity is associated with lower arterial stiffness in older adults: results of the SAPALDIA 3 Cohort Study. Eur J Epidemiol.

[26] Scuteri A, Brancati AM, Gianni W, Assisi A, Volpe M (2005). Arterial stiffness is an independent risk factor for cognitive impairment in the elderly: a pilot study. J Hypertens.

[27] Fukuhara M, Matsumura K, Ansai T, Takata Y, Sonoki K, Akifusa S (2006). Prediction of cognitive function by arterial stiffness in the very elderly. Circ J.

[28] Watson NL, Sutton-Tyrrell K, Rosano C, Boudreau RM, Hardy SE, Simonsick EM (2011). Arterial stiffness and cognitive decline in well-functioning older adults. J Gerontol A Biol Sci Med Sci.

[29] Hanon O, Haulon S, Lenoir H, Seux ML, Rigaud AS, Safar M (2005). Relationship between arterial stiffness and cognitive function in elderly subjects with complaints of memory loss. Stroke.

[30] Flammer AJ, Anderson T, Celermajer DS, Creager MA, Deanfield J, Ganz P (2012). The assessment of endothelial function: from research into clinical practice. Circulation.

[31] Avolio A (2013). Arterial stiffness. Pulse (Basel).

[32] Laurent S, Boutouyrie P (2020). Arterial stiffness and hypertension in the elderly. Front Cardiovasc Med.

[33] Shirwany NA, Zou MH (2010). Arterial stiffness: a brief review. Acta Pharmacol Sin.

[34] Townsend RR, Wilkinson IB, Schiffrin EL, Avolio AP, Chirinos JA, Cockcroft JR (2015). Recommendations for improving and standardizing vascular research on arterial stiffness: a scientific statement from the american heart association. Hypertension (Dallas, Tex: 1979).

[35] Green DJ, Jones H, Thijssen D, Cable NT, Atkinson G (2011). Flow-mediated dilation and cardiovascular event prediction: does nitric oxide matter?. Hypertension (Dallas, Tex: 1979).

[36] Benjamin EJ, Larson MG, Keyes MJ, Mitchell GF, Vasan RS, Keaney JF (2004). Clinical correlates and heritability of flow-mediated dilation in the community: the Framingham heart study. Circulation.

[37] Hamburg NM, Palmisano J, Larson MG, Sullivan LM, Lehman BT, Vasan RS (2011). Relation of brachial and digital measures of vascular function in the community: the Framingham heart study. Hypertension (Dallas, Tex: 1979).

[38] Mitchell GF, Parise H, Benjamin EJ, Larson MG, Keyes MJ, Vita JA (2004). Changes in arterial stiffness and wave reflection with advancing age in healthy men and women: the Framingham heart study. Hypertension (Dallas, Tex: 1979).

[39] Lakatta EG, Levy D (2003). Arterial and cardiac aging: major shareholders in cardiovascular disease enterprises: part I: aging arteries: a “set up” for vascular disease. Circulation.

[40] Lakatta EG, Levy D (2003). Arterial and cardiac aging: major shareholders in cardiovascular disease enterprises: part II: the aging heart in health: links to heart disease. Circulation.

[41] Laurent S, Cockcroft J, Van Bortel L, Boutouyrie P, Giannattasio C, Hayoz D (2006). Expert consensus document on arterial stiffness: methodological issues and clinical applications. Eur Heart J.

[42] Thijssen DHJ, Bruno RM, van Mil A, Holder SM, Faita F, Greyling A (2019). Expert consensus and evidence-based recommendations for the assessment of flow-mediated dilation in humans. Eur Heart J.

[43] Determinants of pulse wave velocity in healthy people and in the presence of cardiovascular risk factors: ‘establishing normal and reference values’. Eur Heart J. 2010;31(19):2338–50. 10.1093/eurheartj/ehq165 PMC2948201 20530030

[44] Sugawara J, Hayashi K, Yokoi T, Cortez-Cooper MY, DeVan AE, Anton MA (2005). Brachial-ankle pulse wave velocity: an index of central arterial stiffness?. J Hum Hypertens.

[45] Tanaka H, Munakata M, Kawano Y, Ohishi M, Shoji T, Sugawara J (2009). Comparison between carotid-femoral and brachial-ankle pulse wave velocity as measures of arterial stiffness. J Hypertens.

[46] Zhang Y, Agnoletti D, Xu Y, Wang JG, Blacher J, Safar ME (2014). Carotid-femoral pulse wave velocity in the elderly. J Hypertens.

[47] Inaba Y, Chen JA, Bergmann SR (2010). Prediction of future cardiovascular outcomes by flow-mediated vasodilatation of brachial artery: a meta-analysis. Int J Cardiovasc Imaging.

[48] Matsuzawa Y, Kwon TG, Lennon RJ, Lerman LO, Lerman A (2015). Prognostic value of flow-mediated vasodilation in brachial artery and fingertip artery for cardiovascular events: a systematic review and meta-analysis. J Am Heart Assoc.

[49] Ras RT, Streppel MT, Draijer R, Zock PL (2013). Flow-mediated dilation and cardiovascular risk prediction: a systematic review with meta-analysis. Int J Cardiol.

[50] Xu Y, Arora RC, Hiebert BM, Lerner B, Szwajcer A, McDonald K (2014). Non-invasive endothelial function testing and the risk of adverse outcomes: a systematic review and meta-analysis. Eur Heart J Cardiovasc Imaging.

[51] Vlachopoulos C, Aznaouridis K, Stefanadis C (2010). Prediction of cardiovascular events and all-cause mortality with arterial stiffness: a systematic review and meta-analysis. J Am Coll Cardiol.

[52] Vlachopoulos C, Aznaouridis K, Terentes-Printzios D, Ioakeimidis N, Stefanadis C (2012). Prediction of cardiovascular events and all-cause mortality with brachial-ankle elasticity index: a systematic review and meta-analysis. Hypertension (Dallas, Tex: 1979).

[53] Malachias MVB (2016). 7th Brazilian guideline of arterial hypertension: presentation. Arq Bras Cardiol.

[54] Simão AF, Précoma DB, Andrade JP, Correa Filho H, Saraiva JF, Oliveira GM (2014). I cardiovascular prevention guideline of the Brazilian Society of Cardiology - executive summary. Arq Bras Cardiol.

[55] Green DJ, Hopman MT, Padilla J, Laughlin MH, Thijssen DH (2017). Vascular adaptation to exercise in humans: role of hemodynamic stimuli. Physiol Rev.

[56] Arnett DK, Blumenthal RS, Albert MA, Buroker AB, Goldberger ZD, Hahn EJ (2019). 2019 ACC/AHA Guideline on the Primary Prevention of Cardiovascular Disease: a report of the American College of Cardiology/American Heart Association Task Force on Clinical Practice Guidelines. Circulation.

[57] Pelliccia A, Sharma S, Gati S, Bäck M, Börjesson M, Caselli S (2021). 2020 ESC Guidelines on sports cardiology and exercise in patients with cardiovascular disease. Eur Heart J.

[58] Crichton GE, Elias MF, Robbins MA (2014). Cardiovascular health and arterial stiffness: the Maine-Syracuse Longitudinal Study. J Hum Hypertens.

[59] Ribeiro AL, Duncan BB, Brant LC, Lotufo PA, Mill JG, Barreto SM (2016). Cardiovascular health in Brazil: trends and perspectives. Circulation.

[60] Cornelissen VA, Smart NA (2013). Exercise training for blood pressure: a systematic review and meta-analysis. J Am Heart Assoc.

[61] Soares-Miranda L, Siscovick DS, Psaty BM, Longstreth WT, Mozaffarian D (2016). Physical activity and risk of coronary heart disease and stroke in older adults: the cardiovascular health study. Circulation.

[62] Garber CE, Blissmer B, Deschenes MR, Franklin BA, Lamonte MJ, Lee IM (2011). American College of Sports Medicine position stand. Quantity and quality of exercise for developing and maintaining cardiorespiratory, musculoskeletal, and neuromotor fitness in apparently healthy adults: guidance for prescribing exercise. Med Sci Sports Exerc.

[63] Santos LP, Umpierre D (2020). Exercise, cardiovascular health, and risk factors for atherosclerosis: a narrative review on these complex relationships and caveats of literature. Front Physiol.

[64] Joyner MJ, Green DJ (2009). Exercise protects the cardiovascular system: effects beyond traditional risk factors. J Physiol.

[65] Shiotsu Y, Watanabe Y, Tujii S, Yanagita M (2018). Effect of exercise order of combined aerobic and resistance training on arterial stiffness in older men. Exp Gerontol.

[66] Figueroa A, Okamoto T, Jaime SJ, Fahs CA (2019). Impact of high- and low-intensity resistance training on arterial stiffness and blood pressure in adults across the lifespan: a review. Pflugers Arch.

[67] Li Y, Hanssen H, Cordes M, Rossmeissl A, Endes S, Schmidt-Trucksäss A (2015). Aerobic, resistance and combined exercise training on arterial stiffness in normotensive and hypertensive adults: a review. Eur J Sport Sci.

[68] Son Y, Kim K, Jeon S, Kang M, Lee S, Park Y (2017). Effect of exercise intervention on flow-mediated dilation in overweight and obese adults: meta-analysis. Int J Vasc Med.

[69] Zhang Y, Qi L, Xu L, Sun X, Liu W, Zhou S (2018). Effects of exercise modalities on central hemodynamics, arterial stiffness and cardiac function in cardiovascular disease: systematic review and meta-analysis of randomized controlled trials. PLoS One.

[70] Collier SR, Frechette V, Sandberg K, Schafer P, Ji H, Smulyan H (2011). Sex differences in resting hemodynamics and arterial stiffness following 4 weeks of resistance versus aerobic exercise training in individuals with pre-hypertension to stage 1 hypertension. Biol Sex Differ.

[71] DeVallance E, Fournier S, Lemaster K, Moore C, Asano S, Bonner D (2016). The effects of resistance exercise training on arterial stiffness in metabolic syndrome. Eur J Appl Physiol.

[72] Fernandez-del-Valle M, Gonzales JU, Kloiber S, Mitra S, Klingensmith J, Larumbe-Zabala E (2018). Effects of resistance training on MRI-derived epicardial fat volume and arterial stiffness in women with obesity: a randomized pilot study. Eur J Appl Physiol.

[73] Rakobowchuk M, McGowan CL, de Groot PC, Bruinsma D, Hartman JW, Phillips SM (2005). Effect of whole body resistance training on arterial compliance in young men. Exp Physiol.

[74] Casey DP, Pierce GL, Howe KS, Mering MC, Braith RW (2007). Effect of resistance training on arterial wave reflection and brachial artery reactivity in normotensive postmenopausal women. Eur J Appl Physiol.

[75] Casey DP, Beck DT, Braith RW (2007). Progressive resistance training without volume increases does not alter arterial stiffness and aortic wave reflection. Exp Biol Med (Maywood, NJ).

[76] Miyachi M, Kawano H, Sugawara J, Takahashi K, Hayashi K, Yamazaki K (2004). Unfavorable effects of resistance training on central arterial compliance: a randomized intervention study. Circulation.

[77] Collier SR, Kanaley JA, Carhart R, Frechette V, Tobin MM, Hall AK (2008). Effect of 4 weeks of aerobic or resistance exercise training on arterial stiffness, blood flow and blood pressure in pre- and stage-1 hypertensives. J Hum Hypertens.

[78] Pedralli ML, Eibel B, Waclawovsky G, Schaun MI, Nisa-Castro-Neto W, Umpierre D (2018). Effects of exercise training on endothelial function in individuals with hypertension: a systematic review with meta-analysis. J Am Soc Hypertens.

[79] Pedralli ML, Marschner RA, Kollet DP, Neto SG, Eibel B, Tanaka H (2020). Different exercise training modalities produce similar endothelial function improvements in individuals with prehypertension or hypertension: a randomized clinical trial Exercise, endothelium and blood pressure. Sci Rep.

[80] Ashor AW, Lara J, Siervo M, Celis-Morales C, Oggioni C, Jakovljevic DG (2015). Exercise modalities and endothelial function: a systematic review and dose-response meta-analysis of randomized controlled trials. Sports Med (Auckland, NZ).

[81] Silva J, Menêses AL, Parmenter BJ, Ritti-Dias RM, Farah BQ (2021). Effects of resistance training on endothelial function: a systematic review and meta-analysis. Atherosclerosis.

[82] Lopes S, Afreixo V, Teixeira M, Garcia C, Leitão C, Gouveia M (2021). Exercise training reduces arterial stiffness in adults with hypertension: a systematic review and meta-analysis. J Hypertens.

[83] Ashor AW, Lara J, Siervo M, Celis-Morales C, Mathers JC (2014). Effects of exercise modalities on arterial stiffness and wave reflection: a systematic review and meta-analysis of randomized controlled trials. PLoS One.

[84] Moher D, Liberati A, Tetzlaff J, Altman DG; PRISMA Group. Preferred reporting items for systematic reviews and meta-analyses: the PRISMA statement. BMJ. 2009;339:b2535. 10.1136/bmj.b2535. PMID: 19622551; PMCID: PMC2714657. 10.1136/bmj.b2535 PMC2714657 19622551

[85] Moher D, Shamseer L, Clarke M, Ghersi D, Liberati A, Petticrew M (2015). Preferred reporting items for systematic review and meta-analysis protocols (PRISMA-P) 2015 statement. Syst Rev.

[86] Caspersen CJ, Powell KE, Christenson GM (1985). Physical activity, exercise, and physical fitness: definitions and distinctions for health-related research. Public Health Rep.

[87] USDoHaHS (2018). 2018 Physical activity guidelines advisory committee scientific report. In: Services USDoHaH, editor.

[88] Lee M, Carroll TJ (2007). Cross education: possible mechanisms for the contralateral effects of unilateral resistance training. Sports Med (Auckland, NZ).

[89] Hickson RC (1980). Interference of strength development by simultaneously training for strength and endurance. Eur J Appl Physiol Occup Physiol.

[90] Ferketich AK, Kirby TE, Alway SE (1998). Cardiovascular and muscular adaptations to combined endurance and strength training in elderly women. Acta Physiol Scand.

[91] Robinson KA, Dickersin K (2002). Development of a highly sensitive search strategy for the retrieval of reports of controlled trials using PubMed. Int J Epidemiol.

[92] Glanville J, Foxlee R, Wisniewski S, Noel-Storr A, Edwards M, Dooley G (2019). Translating the Cochrane EMBASE RCT filter from the Ovid interface to Embase.com: a case study. Health Info Libr J.

[93] Green DJ, Maiorana A, O'Driscoll G, Taylor R (2004). Effect of exercise training on endothelium-derived nitric oxide function in humans. J Physiol.

[94] Sterne JAC (2019). RoB 2: a revised tool for assessing risk of bias in randomised trials. BMJ.

[95] Atkins D, Best D, Briss PA, Eccles M, Falck-Ytter Y, Flottorp S (2004). Grading quality of evidence and strength of recommendations. BMJ (Clinical research ed).

[96] Higgins JPT, Cochrane C (2019). Cochrane handbook for systematic reviews of interventions.

[97] IntHout J, Ioannidis JP, Rovers MM, Goeman JJ (2016). Plea for routinely presenting prediction intervals in meta-analysis. BMJ Open.

[98] Dias S, Sutton AJ, Welton NJ, Ades AE (2013). Evidence synthesis for decision making 3: heterogeneity--subgroups, meta-regression, bias, and bias-adjustment. Med Decis Making.

[99] Higgins JPT, Thomas J, Chandler J, Cumpston M, Li T, Page MJ (2019). Cochrane handbook for systematic reviews of interventions version 6.0.

[100] Liberati A, Altman DG, Tetzlaff J, Mulrow C, Gøtzsche PC, Ioannidis JPA (2009). The PRISMA statement for reporting systematic reviews and meta-analyses of studies that evaluate healthcare interventions: explanation and elaboration. BMJ.

[101] Boutron I, Page MJ, Higgins JP, Altman DG, Lundh A, Hróbjartsson A (2019). Considering bias and conflicts of interest among the included studies.

[102] Ceciliato J, Costa EC, Azevêdo L, Sousa JC, Fecchio RY, Brito LC (2020). Effect of resistance training on arterial stiffness in healthy subjects: a systematic review and meta-analysis. Curr Hypertens Rep.

